# Selected Political Criminal Poisonings in the Years 1978–2020: Detection and Treatment

**DOI:** 10.3390/toxics10080468

**Published:** 2022-08-12

**Authors:** Zuzanna Brunka, Jan Ryl, Piotr Brushtulli, Daria Gromala, Grzegorz Walczak, Sonia Zięba, Dorota Pieśniak, Jacek Sein Anand, Marek Wiergowski

**Affiliations:** 1 Student Scientific Society of the Medical University of Gdańsk, M. Skłodowskiej-Curie 3a Str., 80-210 Gdańsk, Poland; 2 Department of Forensic Medicine, Faculty of Medicine, Medical University of Gdańsk, M. Skłodowskiej-Curie 3a Str., 80-210 Gdańsk, Poland; 3 Division of Clinical Toxicology, Faculty of Health Sciences with the Institute of Maritime and Tropical Medicine, Medical University of Gdańsk, M. Skłodowskiej-Curie 3a Str., 80-210 Gdańsk, Poland; 4 Pomeranian Center of Toxicology, Kartuska 4/6 Str., 80-104 Gdańsk, Poland

**Keywords:** criminal poisoning, political intoxication, ricin, fentanyl, TCDD, polonium-210, Novichok, VX

## Abstract

Criminal poisonings are among the least frequently detected crimes in the world. Lack of suspicion of this type of event by police officers and prosecutors, clinical symptoms imitating many somatic diseases and technical difficulties in diagnostics, as well as high research costs make the actual frequency of these events difficult to estimate. The substance used for criminal poisoning is often characterized by: lack of taste, color and smell, delayed action, easy availability and difficulty to detect. The aim of the study was to analyze selected cases of political poisoning that took place in the years 1978–2020, to describe the mechanisms of action of the substances used and to evaluate the diagnosis and treatment. The analyzed cases of criminal poisoning concerned: Georgi Markov (ricin), Khalid Maszal (fentanyl), Wiktor Yushchenko (TCDD dioxin), Jasir Arafat (polonium ^210^Po isotope), Alexander Litvinenko (polonium ^210^Po isotope), Kim Jong-Nam (VX), Sergei Skripal (Novichok) and Alexei Navalny (Novichok). Contemporary poisons, to a greater extent than in the past, are based on the use of synthetic substances from the group of organophosphorus compounds and radioactive substances. The possibility of taking appropriate and effective treatment in such cases is the result of many factors, including the possibility of quick and competent rescue intervention, quick and reliable detection of the toxic substance and the possibility of using an antidote.

## 1. Introduction

Toxicology still refers back to the words of Paracelsus, who stated that toxic effect of each substance depends mostly on its dose. Besides the dose, the toxicity is affected by factors reliant directly on the poisonous substance, such as the route and rate of administration, hydro- and lipophilicity, physical state and formulation (e.g., liquid, gas, solid) and the clinical state of the victim, particularly including: age, sex, body mass, comorbidities and genetic predisposition. The poison used in a criminal poisoning should be characterized by such features as: colorlessness, lack of taste and smell, delayed toxic effects, difficulties in detection and easy availability (Table 1) [1].

Today, with the development of diagnostic techniques, the chance of revealing various types of criminal poisoning has increased. However, it is worth realizing that at the same time there has also been significant progress in planning criminal activities.

## 2. Criminal Poisoning Cases

We would like to outline the historical background of politically motivated crime poisonings, with an emphasis primarily on the detection of toxic substances and medical treatment. There are many poisonings in which circumstances are of a political nature but no toxic substances or their metabolites have been detected. One such example is the case of the suspected poisoning of Pyotr Verzilov, Pussy Riot activist [3]. Verzilov fell ill on 11 September 2018 in Moscow. He lost his eyesight and ability to speak, became delirious and lost consciousness. He was hospitalized in Moscow in critical condition and four days later Verzilov was flown to Berlin [4]. Staff of the Berlin Charité hospital believed that, although there were no traces of poison in Verzilov’s system, there was no other explanation for his condition [5].

Table 2 describes selected cases of politically motivated poisonings in the years 1978–2020, with particular emphasis on the circumstances of the event, symptoms of poisoning and the undertaken treatment.

The common feature of the poisoning cases described above was the alleged or proven involvement of secret services in the physical elimination of the political opponents. The symptoms were experienced by the victims almost immediately after poisoning (fentanyl, VX) or a few hours after exposure (ricin, Novichok, polonium ^210^Po isotope). The following describes the toxicological properties of selected toxic substances used for criminal purposes, as well as the diagnostic methods and the proposed treatment methods.

## 3. Toxicological Properties, Treatment and Diagnostics

The physicochemical and toxicological properties for selected criminal poisons are presented in Table 3 [16]. The most important factors determining the potency of the toxic effect were the dose and route of administration. It is worth noting that there is a very limited amount of data on poisons’ doses that are lethal to humans [17].

### 3.1. Ricin

#### 3.1.1. Properties, Metabolic Pathway and Toxic Effects

Ricin is a protein found in a plant called Ricinus communis. It is one of the first lectins detected, i.e., proteins that nonenzymatically attach to membrane sugar receptors. The biological properties of ricin were first described in 1888 by Hermann Stilmark [29]. Ricin is classified as an extremely hazardous substance [30,31]. However, production of the ricin is difficult to legal control because the castor bean plant from which ricin is derived can be grown at home without any special care. In the US, scientists must register it in the Department of Health and Human Services to use ricin and investigators possessing less than 1 g are exempt from regulation [32]. Castor bean seeds, in addition to ricin itself, also contain a homologous but much less toxic agglutinin called RCA120. Ricin is composed of two RTA and RTB protein chains (having 267 and 262 amino acids, respectively), linked by a disulfide bridge. RCA120, in turn, is a protein composed of four chains, 2 RTA and 2 RTB, linked by a disulfide bridge between the A chains. Both compounds show a very high sequence homology of their amino acids. However, ricin is a potential toxic substance, while RCA120 exhibits strong hemagglutinating properties.

The RTB chain is responsible for binding with galactose. The RTB chain is responsible for the entry of ricin into the cell, which is achieved by the production of endosomes. Some of the chains are transported to the Golgi apparatus. Others to the endoplasmic reticulum, in which the RTB is detached from the endosome protein, and the RTA chain itself passes into the cytosol of the cell using the ERAD (ER-associated degradation) pathway. The RTA chain is an enzyme (RNA N-glycosidase) and is responsible for ribosome inactivation (RIP—ribosome inactivating protein). Its action is based on the removal of adenine rRNA from 28S, which prevents the attachment of the translational factor EF2 (elongation factor-2) to the ribosome. In this way, protein synthesis is inhibited, which ultimately leads to cell death [33].

Ricin poisoning is characterized by a delayed onset of symptoms and a slow action with fatal outcome. The lethal dose of ricin depends on the route of its administration (Table 3). The in vivo toxicity of the substance for humans after oral administration is 1–20 mg/kg body weight, while when administered by injection or inhalation, this dose may be slightly lower [34].

After ingestion of the toxin through the alimentary tract, nausea, vomiting, diarrhea and abdominal pain occur. Within about 4–36 h, symptoms may progress, accompanied by: arterial hypertension, renal failure and liver damage.

In the case of inhalation, symptoms usually develop within about 8 h and include: cough, shortness of breath, joint pain and fever. Occasionally, these types of patients suffer from respiratory distress and death.

In the case of injection, local swelling and redness appear at the site of injection of the toxin. The first symptoms of poisoning develop within approx. 6 h. Among them, the dominant ones are: weakness and muscle pain. After the next 24–36 h, symptoms progress, including: nausea, fever and hypotension, as well as multiorgan failure or death.

#### 3.1.2. First Aid and Treatment

Treatment of ricin poisoning is symptomatic. It includes the intravenous administration of fluids and vasopressors. In case of oral poisoning, it is possible to administer activated charcoal. Gastric lavage is considered only in cases where the intoxicity has occurred no more than one hour earlier.

Currently, the greatest hopes in the postexposure treatment of ricin poisoning are placed in the use of neutralizing antibodies. So far, two preparations for vaccination have been tested: ricin chain deactivated with formaldehyde (ricin toxoid) and deglycosylated ricin. Research using genetic recombination methods has led to the production of the RiVax vaccine.

Animal studies were carried out, including pigs exposed to lethal pulmonary exposure and systemic ricin with the use of anti-ricin F(ab’)2 antibodies of equine origin. The results of this study showed that it is possible to create postexposure remedies for ricin poisoning in humans [35].

#### 3.1.3. Diagnostics

Diagnosis of ricin poisoning is based on history, identification of ricin in biological fluids or environmental samples. In clinical samples, ricin is difficult to detect due to its strong binding to glycosylated protein. Free ricin (with a molecular weight of 164 Da) is a marker of castor bean consumption [33]. There are analytical methods that enable the identification of ricin in blood, urine and in the vitreous humor of the eye (including ELISA tests, liquid chromatography with LC-MS/MS tandem mass spectrometry). Unfortunately, the level of ricin in body fluids does not have to correlate with the severity of symptoms.

### 3.2. Fentanyl

#### 3.2.1. Properties, Metabolic Pathway and Toxic Effects

Fentanyl is a synthetic compound that belongs to the opioid family. First opioid substance was isolated in 1806 by Serturner which, as a tribute to the god of sleep Morpheus, was named morphine. Because of Serturner’s strong addiction to this substance that he developed soon after, he managed to describe the consequences of its chronic abuse in great detail. Since the time of their discovery, opioids have come to be an essential drug in everyday practice of medicine, mainly as analgesics. However, due to their strong addictive potential, they have also been used widely as recreational drugs.

Fentanyl is one of the most potent opioids, being a 100 times more potent than morphine. As a strongly lipophilic substance it enters the tissue compartments with ease (especially the central nervous system) and clinically produces an opioid toxidrome with a very characteristic presentation: bradycardia, bradypnea, hypotonia [36]. Fentanyl is controlled psychoactive substance by drug law in many countries (e.g., in UK, US, Netherlands, Poland, Canada) [37] and is also applied in medicine (only by prescription) [38]. However deaths involving synthetic opioids other than methadone (primarily fentanyl) continued to rise in the US in 2015–2020 [39]. In developed countries, the illicit market for the supply of fentanyl and related substances is very large [40].

On a molecular level fentanyl acts as a μ, δ and κ receptor agonist, each of which is coupled with a Gi protein (it causes a decrease in intracellular cAMP levels). Fentanyl’s interaction with an opioid receptor causes a decrease in calcium influx in the presynaptic neuron, which then suppresses the release of neurotransmitters into the synaptic cleft, as well as hyperpolarization of postsynaptic neuron due to potassium ion the efflux. All these effects impede physiological neuronal transmission [41].

**Figure 1:**
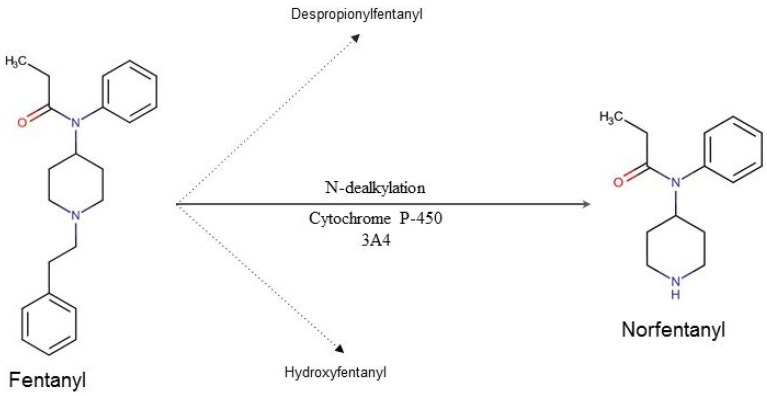
Fentanyl’s metabolic pathway in humans. Ninety-nine percent of the metabolized fentanyl is converted into norfentanyl. The remaining 1% is converted to despropionyl fentanyl and hydroxyfentanyl.

The most important organ in fentanyl metabolism is the liver (Figure 1). The first phase of its biotransformation is conducted by CYP3A4, also present in enterocytes, which determines its particularly strong first pass effect. During the second phase fentanyl’s metabolites are being conjugated with the glucuronic acid, after which they are eliminated with the urine.

#### 3.2.2. First Aid and Treatment

**Figure 2:**
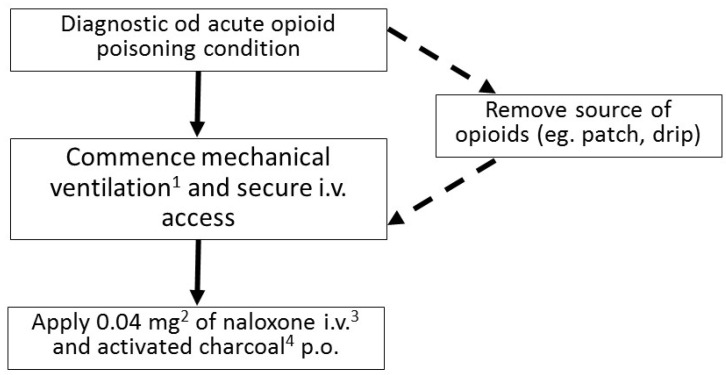
Flowchart describing the management of acute opioid poisoning in an adult (^1^ Most commonly noninvasive; if the GCS < 8 endotracheal intubation is indicated. ^2^ If no improvement occurs after 2–3 min the dose can be increased first to 0.5 mg, then, after another 2–3 min, to 2 mg, then to 4 mg, 10 mg, and up to the maximum dose of 15 mg. ^3^ If securing an i.v. access is impossible, naloxone can be administered i.m. or intranasally. ^4^ Recommended in absence of contraindications).

In spite of a fairly characteristic clinical presentation of an opioid toxidrome, fentanyl intoxication may be sometimes difficult to diagnose, even for an experienced clinician (Figure 2). The most important steps in the treatment are: securing patient’s airways, administering the antidote—naloxone—and providing ventilation support if needed. Naloxone is administered in fractioned doses, with the first one being usually 0.4 mg. After administering naloxone, the patient’s condition should be monitored closely for 2–3 min, and in the absence of improvement, the dose should be increased in a gradual way. Special care must be taken when administering naloxone in patients with an opioid addiction. Overdosing naloxone in such individuals may cause a severe opioid withdrawal syndrome, which can be life-threatening.

#### 3.2.3. Diagnostics

Owing to the fact that fentanyl is a compound, in which metabolites are excreted primarily with urine, urine tests are going to be the base of an accurate detection of this substance. This can be a simple immunochemical test (strip test), as well as more advanced and sensitive diagnostic techniques such as gas chromatography with mass spectrometry (GC/MS) or liquid chromatography with tandem mass spectrometry (LC–MS/MS). It is also possible to detect fentanyl in an environmental sample for instance via ion mobility spectrometry (IMS), which can detect even a nanograms of this substance in a sample tested [36].

### 3.3. TCDD

#### 3.3.1. Properties, Metabolic Pathway and Toxic Effects

TCDD (2,3,7,8-tetrachlorodibenzo-*p*-dioxin) is the most representative, best-known and most toxic substance from the group of dioxin-like compounds (DLCs). Dioxins were discovered in 1957 during a veterinary investigation to explain the mass extinction of farmed chickens. The most scientific reports on the impact of DLCs on living organisms appeared in the 1970s during the Vietnam War [42].

Pure dioxins are colorless, crystalline solids, insoluble in water and well soluble in organic solvents. About 70% of the world’s TCDD production comes from the controlled incineration of municipal waste. The main source of population exposure is the consumption of fatty animal products. Exposure to high concentrations of TCDD as a by-product during work (production of fertilizers, pesticides, agent yellow sprays, etc.), industrial accidents and disasters are known. Several cases of the use of TCDD as a poison used for criminal purposes have also been described.

TCDD, like other dioxins, is subject to legal regulations concerning food safety and environmental protection. For example, The United States Environmental Protection Agency (EPA) established an oral reference dose of 0.7 pg TCDD/kg b.w. per day [43]. Pure TCDD has never been produced commercially except for scientific research application [44].

Bioavailability of TCDD after p.o. administration ranges from 2 to 42% (highest values for the oil carrier). It undergoes little first-pass metabolism. DLCs in the blood are mainly carried by plasma lipoproteins. In the first hours, accumulation is mainly observed in the liver, and after a week, mainly in adipose tissue.

In the liver, DLCs undergo oxidation and hydroxylation, followed by a coupling reaction with glucuronic acid or glutathione (the formation of products that can be removed in the urine). The parent compound and its metabolites are gradually eliminated in the feces (90% of total elimination). DLC metabolites are also excreted in urine, sweat and milk, albeit to a lesser extent. The half-life in the human body is approx. 5–10 years, and its length is inversely proportional to the time of exposure and exposure. Elimination is according to first order kinetics [45].

The main toxic effect of dioxins is direct genotoxicity. Less common, but more important in the case of exposure to high concentrations (as in the case of criminal poisoning), is the activation of the aryl hydrocarbon receptor pathway [46]. The effects of this activation are: cellular oxidative stress, alteration of gene expression, activation of the inflammatory response, dysfunction of mitochondrial membranes, disturbances in the microstructure and function of almost every organ. Moderate granulocytosis, insulin resistance and an increase in lipid levels are also observed. In humans, exposure to dioxins significantly increases the risk of cancer development and also reduces fertility. Additionally, it may develop: diabetes mellitus, cirrhosis of the liver and hypothyroidism. A very characteristic but late symptom (3–10 weeks from the time of poisoning or exposure) is the occurrence of chloracne [47].

#### 3.3.2. First aid and Treatment

Currently, there are no generally recognized principles of treating acute TCDD poisoning. Medical activities boil down to the treatment of hepatic failure, the proper nutrition of the patient and pain therapy. In order to alleviate the course of the acute phase and, in particular, to avoid long-term complications, the priority is the rapid elimination of the poison. This process can be accelerated by the oral administration of nonabsorbed lipid substitutes as well as prokinetic and choleretic drugs. Glutathione supplementation and, in the long term, fat loss may also prove beneficial. Lipid-lowering treatment, including LDL-apheresis and antioxidant activity using tocopherol, did not show high effectiveness [48].

#### 3.3.3. Diagnostics

In the case of poisoning by Viktor Yushchenko, the levels of TCDD concentrations in blood serum were determined chromatographically, and it was shown that they were over 50,000 times higher than in the general population. The identification and quantification of TCDD and its metabolites in biological material (serum, fatty tissue, feces, skin, urine and sweat) was monitored for 3 years using liquid–liquid extraction and gas chromatography coupled with high-resolution mass spectrometry. In the preliminary stage, the patient’s feces were freeze-dried and frozen after homogenization. The adipose tissue and skin samples were frozen in liquid nitrogen and then homogenized by grinding (with a pestle and mortar). Blood, urine and sweat were prefrozen. Sorg et al. suggest that to monitor poisoning over a longer period of time, apart from TCDD, the concentration levels of its metabolites should be determined—2,3,7-trichloro-8-hydroxydibenzo-pdioxin and 1,3,7,8-tetrachloro-2-hydroxydibenzo-p-dioxin [49].

### 3.4. Isotope of Polonium ^210^Po

#### 3.4.1. Properties, Metabolic Pathway and Toxic Effects

Polonium is a rare metal with high radioactivity and a short half-life (138 days). The biological half-life in the human body is approximately 40 days. The only natural isotope is polonium-210, which is found in trace amounts in uranium ores. Polonium-210 emits high-energy α particles, and the product of its decay is lead isotope ^206^Pb. During the decay, an energy of 5.3 MeV is emitted. ^210^Po dissolves in aqueous solutions because it forms simple salts in dilute acids [50,51]. About 0.5 g of polonium is self-heating to reach a temperature above 500 °C. The metabolic pathway of polonium-210 is unknown due to the lack of suitable analogues [52].

Polonium 210 is an element with a wide range of industrial applications and is relatively easily accessible with little regulations [53]. Only about 100 g of polonium-210 is produced worldwide each year, and licensed distributors import a very small amount into the US. In daily life the chance of someone getting hold of a lethal dose of polonium-210 is very small [54]. Polonium-210 and its compounds should be kept in a double compartment. In addition, neoprene gloves should be used to protect against radiation better than rubber gloves [55].

The toxicity of polonium-210 results from its high radioactivity (specific activity is 166 TBq/g). The emitted alpha particles have a range of about 40–50 mm in biological tissue, as they are retained by the *stratum corneum*. Therefore, the absorption of this element through the skin does not pose a major threat, and in the event of exposure, remove clothing and wash thoroughly. Poisoning occurs when ingested orally, through open wounds or as a result of breathing contaminated air. When polonium-210 is orally ingested, it easily enters the bloodstream. It is mainly deposited in soft tissues. Its highest concentration is recorded in the liver, spleen, bone marrow, kidneys and skin [51].

#### 3.4.2. First aid and Treatment

Jefferson proposed an aid and treatment regimen after poisoning with polonium-210 by the oral route [51], in which the most important steps are: gastric lavage (up to an hour after ingestion), use of antiemetic drugs, intravenous administration of fluids and analgesics, treatment bone marrow failure, chelation therapy and palliative care (see Table 4 for detailed treatment steps) [56].

#### 3.4.3. Diagnostics

If a patient develops nausea, vomiting and bloody diarrhea of unknown etiology, radiation sickness should be considered. Additional symptoms that may be suggested by the above-mentioned the disease include: bleeding, hair loss, lymphopenia (especially without elevated temperature), low number of thrombocytes and leukocytes—occurring after a few days or weeks after gastrointestinal symptoms. Diagnostics are based primarily on chromosome analysis, which can help determine the effect of radiation on the body and estimate its dose. Urine and feces are also cultured to detect the presence of **^210^**Po and to exclude other etiological factors [51].

### 3.5. VX

#### 3.5.1. Properties, Metabolic Pathway and Toxic Effects

VX (Venomous agent X) belongs to the group of V class paralytic and convulsive agents, which are difficult to evaporate liquids [28,57]. VX is considered a weapon of mass destruction. Its production and storage (over 100 g per year) is prohibited by Chemical Weapons Convention. These substances can only be used in medicine, pharmacy and research [58].

**Figure 3:**
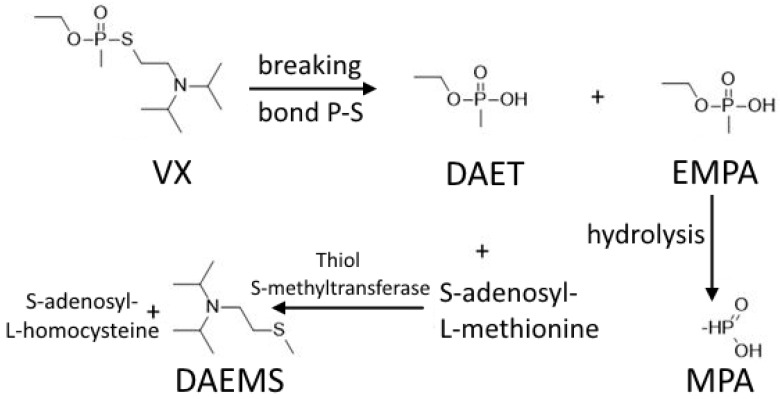
VX metabolic pathway in the human body. VX: N-[2-[ethoxy(methyl)phosphoryl]sulfonylethyl]-N-propan-2-ylpropan-2-amine, DAET: 2-(diisopropylamino)ethanethiol, EMPA: ethylmethylphosphonic acid, MPA: ethylmethylphosphonic acid, DAEMS: 2-(diisopropylaminoethyl)methylsulfide [59].

Pharmacodynamically, it blocks the activity of cholinesterases with a “senescence” time of binding (dealkylation of VX bound to cholinesterase, which permanently inactivates the enzyme) up to 48 h [17]. Additionally, it increases the concentration of the enzyme inducible nitric oxide synthase (iNOS), activation of xanthine oxidoreductase, enhances the expression of IL-6 and decreases the content of surfactant D in the lung tissue of the tested animals [57,59]. The VX metabolic pathway is shown in Figure 3.

Contact with VX causes the appearance of cholinergic toxidrome. It can induce immediate bronchoconstriction or ARDS in the lung spaces. VX crosses tissue barriers (from respiratory to digestive tract), causing intestinal symptoms [60,61].

#### 3.5.2. First Aid and Treatment

**Figure 4:**
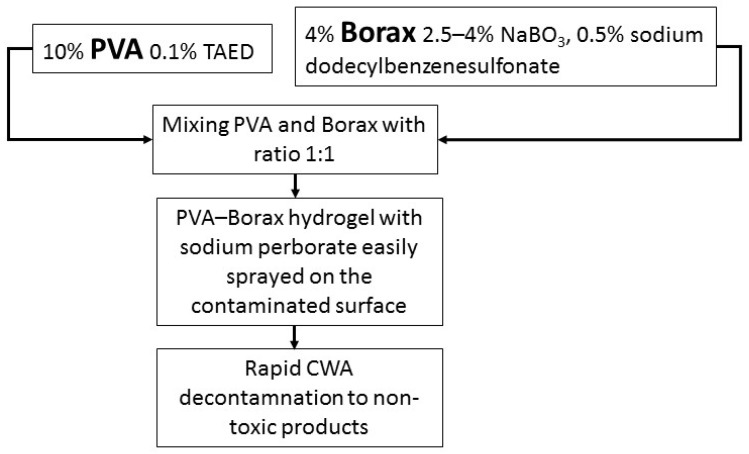
Scheme of neutralization and treatment after VX contamination with PVA-Borax hydrogel. (PVA—polyvinyl alcohol, TAED—tetraacetyl ethylenediamine, Borax—sodium tetraborate) [62].

The main recommendation of help is to limit contact with the toxin. Contaminated clothing should be removed as soon as possible and the body thoroughly washed with water or soap and water. It is proposed to use mixtures of substances with cleansing and neutralizing properties, e.g., PVA-Borax hydrogel with the addition of catalysts (Figure 4) [62]. If decontamination is temporarily not possible, lowering the ambient temperature slows the penetration of the toxin through the skin (at −15 °C effectively up to 1 h; further the absorption accelerates and an hour later exceeds the values for 20 °C) [63]. If swallowed, do not induce vomiting [64].

It is estimated that the above mixture decomposes approx. 50% of VX in 30 min (100% in 3.5 h). Neutralization takes place by peroxide hydrolysis in an environment with a pH of ~9, with HOO- ions (derived from NaBO3) and oxidation with peracetic acid (from TAED). The end product of the reaction is only safe EMPA (ethylmethylphosphonic acid). The key role of NaBO_3_ was noted on this occasion, as in its absence the reaction was slower and with the formation of desethyl-VX. The hydrogel adheres well to various surfaces—in 5 min it adjusts the shape to the applied structure, stiffening after 10 min.

Treatment of poisoning with paralytic and convulsive factors in adults, according to Polish and American standards, is slightly different, but it is absolutely necessary to include atropine and one of the oximes as soon as possible—this combination effectively reduces mortality and long-term effects poisoning (Table 5) [65,66,67,68].

#### 3.5.3. Diagnostics

The first step in the diagnosis of poisoning with organophosphorus compounds is the determination of cholinesterases (preferably BuCHE, then ACHE) and albumin with phosphorylated tyrosine-411 in the patient’s blood [69]. The time to decrease and return to normal for the above markers is as follows: BuCHE—minutes to hours, several weeks; ACHE—a few days, 1–3 months; albumin in an animal model detected up to one week [70,71].

**Figure 5:**
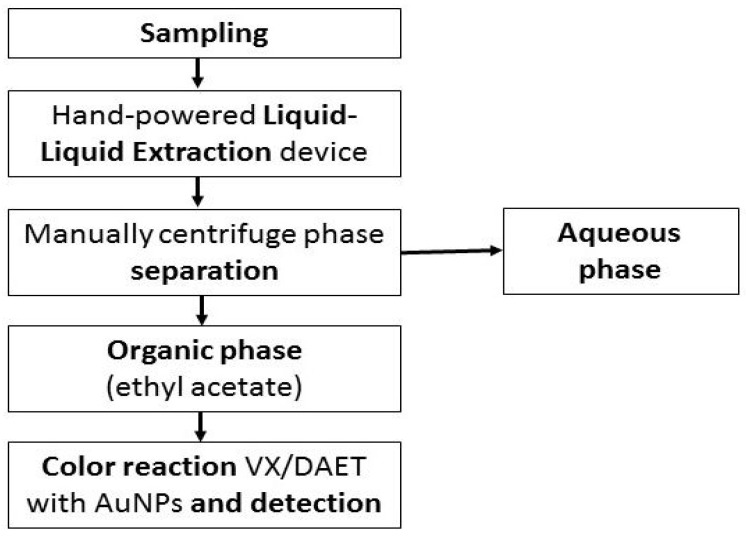
Scheme of colorimetric DAET/VX determination with AuNPs [19].

VX can be specifically detected and determined from blood or urine by commonly used methods such as colorimetry and mass spectrometry (MS) [70]. When MS is used, the detection limit of the analysis is 5 ng/mL for DAEMS and 1 ng/mL for EMPA [16,72]. EMPA and MPA determination is recommended as the DAEMS concentration becomes undetectable several hours after VX exposure. Combat gas detector kits and mobile kits for the rapid determination of cholinesterase levels are also widely used [73,74]. A simple method of colorimetric determination of VX with the use of AuNPs gold nanoparticles should also be mentioned (Figure 5) [70].

The reaction takes place in an acidic environment, where the citric acid acts as a surfactant on the metal surface (while electrostatic interactions are preserved). DAET displaces it, forming a thiol-Au bond. Replacing the citric acid molecule with DAET abolishes the electrostatic interactions and brings the gold particles close enough to each other that van der Waals forces begin to act, which stabilize larger metal clusters. One of the physical properties of AuNPs is the change in color depending on the degree of aggregation, which makes it possible to read the result of a chemical reaction with the naked eye. Metal particles in a slightly acidic environment can be prepared from commonly available reagents, and the obtained solution has a shelf life of 2 weeks. The result is available after a few minutes—if positive, the solution changes its color from red (absorption spectrum 520 nm) to blue (680 nm). The sensitivity of the method for the determination of DAET is 16 ng/mL (100 nM). Due to a special protocol for manual extraction, impurities were eliminated, and the method was highly effective under simulated environmental conditions [64,68].

### 3.6. Novichok

#### 3.6.1. Properties, Metabolic Pathway and Toxic Effects

Organophosphorus compounds in the Novichok group were first synthesized in the 1970s and 1980s in Russia as part of the secret “FOLIANT” program. Novichoks are considered binary compounds (formed from two precursors, with each precursor separately posing no threat) suitable for use as chemical weapons. Novichoks are referred to as fourth-generation CW agents [75]. Due to its high toxicity the Organization for the Prohibition of Chemical Weapons (OPCW) banned the use of Novichok [76].

**Figure 6:**
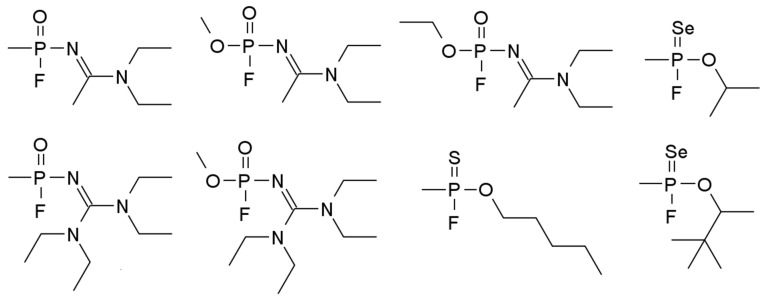
Selected structural formulas of the known Novichoks [75].

According to the available information, the structure of Novichok is based on an organophosphorus core (Figure 6) containing halogen or pseudohalogen substituents—in their chain they contain the -O-N=C(-X)-Y grouping (behind the X and Y can be either chlorine, bromine, fluorine atoms or pseudohalogen groups). These compounds are known to differ from previously known paralyzing agents by their lack of a P-C bond, which allowed them to successfully avoid inclusion in the Chemical Weapons Convention (until 2019) [77].

Novichoks are most commonly found in liquid form. They can also be found in fine powder form. They are extremely stable, which means that they can be stored in containers for long periods of time without changing properties. These compounds do not evaporate and do not dissolve in water [75]. Given the similarity of Novichok to other paralyzing agents, it can be assumed that they decompose when exposed to high pH. The high lipophilicity of these compounds prolongs their detection time in the body. 

There is no official or publicly available information on the toxicity of Novichoks. Experts believe that some of them may be up to 5-8 times more toxic than VX (Table 2) [75].

Their action most likely involves blocking two sites in the active center of acetylcholinesterase [77] and causing irreversible neuropathy by attaching Novichok molecules to sensory endings of peripheral nerves. Inhibition of acetylcholinesterase results in the failure to break down acetylcholine and its continued action on muscarinic and nicotinic receptors, resulting in cholinergic toxidrome [78].

Symptoms resulting from overstimulation of muscarinic receptors include skin flushing, pupil constriction, visual disturbances, drooling and dangerous bronchial hypersecretion, bronchospasm, coughing, dyspnea, lacrimation, sweating, intestinal colic, diarrhea, bradycardia and involuntary urination and defecation. Excessive stimulation of nicotinic receptors is responsible for: tremor, muscle weakness up to complete paralysis, tachycardia, hypertension [79]. It has not been established how long the substance remains toxic—currently it is likely to last from several months to several years.

#### 3.6.2. First Aid and Therapy

**Figure 7:**
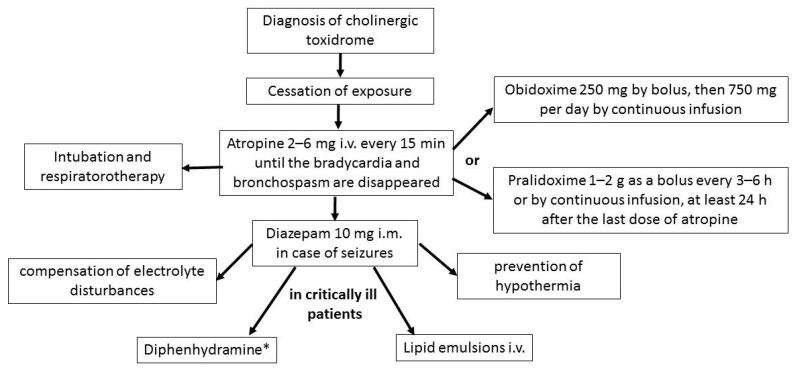
Procedure in the case of suspected Novichok poisoning (*—so far this therapy has been tested only on animals).

In the management of patients who may have been exposed to Novichok, it is important to recognize cholinergic toxidrome promptly, to discontinue exposure and to treat it intensively, even before the results of the directional diagnosis are available [80]. Specific treatment protocols are still lacking, so the same methods are used as for poisoning with other well-known paralyzing agents and those acting through cholinesterase inhibition (Figure 7).

Therapy should begin with immediate intravenous administration of atropine 2–6 mg and repeated doses every 5–10 min until bradycardia, bronchospasm and excessive sweating resolve. Oxime administration, although effective, should not delay the use of atropine. Because of respiratory muscle paralysis, intubation and mechanical ventilation are necessary. If convulsions occur, diazepam 10–20 mg i.v. is used. Continued symptomatic treatment is necessary. In case of bacterial infection, antibiotic therapy should be introduced. The literature also describes the use of diphenhydramine (so far this therapy has been tested only on animals) and intravenous administration of lipid emulsions in critically ill patients [67,78].

#### 3.6.3. Diagnostics

The diagnostics of poisoning with Novichok group compounds is based primarily on the earliest possible diagnosis of cholinergic toxidrome. Recognition of a characteristic symptom complex allows immediate implementation of potentially life-saving treatment; further diagnostics is of secondary importance and is performed to confirm the substance used and possibly more precisely direct the treatment.

Testing the activity of cholinesterase and butyrylcholinesterase (an enzyme that breaks down molecules of paralyzing agents) in blood shows complete blockade of both enzymes, which seems quite obvious considering the nature of action of Novichok—it belongs to the group of cholinesterase inhibitors [75]. Mass spectrometry is a commonly used method that allows detection of both acetylcholinesterase-bound Novichok on erythrocytes and those bound to albumin. However, it should be mentioned here that the mass spectrometry assay shows the highest specificity for Novichok bound to butyrylcholinesterase—according to available reports, Novichok molecules bound to this enzyme are much larger than other known paralyzing agents [81].

In the course of the study, a full toxicological examination should also be performed for known and available poisons that may cause a similar set of symptoms. Neurophysiologic studies performed when in doubt will show typical conduction abnormalities, i.e., neuromuscular plate blockade and variable prolongation of the action potential between the muscle fibers tested, the so-called jitter [80].

## 4. Discussion

**Figure 8:**
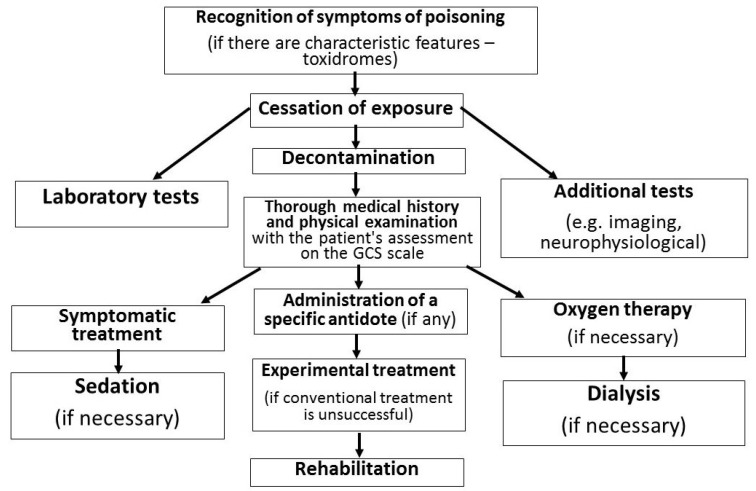
Algorithm for dealing with suspected criminal poisoning.

Due to the large diversity of pharmacokinetic and pharmacodynamic properties of toxic substances used for criminal purposes, it is impossible to apply a single and universal scheme for diagnostics and treatment after poisoning. Nevertheless, the flowchart should take into account at least the stages listed in Figure 8.

### 4.1. Ricin

Ricin is a vegetable protein with a very high toxicity, and, as the case of Georgi Markov shows, it causes great diagnostic and treatment difficulties. There is no detailed information on the human toxicity of this compound nor on effective treatments for ricin poisoning. However, this compound is not toxic enough to be used on a large scale in bioterrorism. Historically, its criminal use has been associated with small attacks or in individual cases. For example, apart from Georgi Markov—it is suspected that ricin was also used in the attack on Vladimir Kostov in the Paris metro 10 days before the attack on Markov. There were also attempts at attacks with the use of ricin by the percutaneous route, among others Castor letters sent to White House. It is also possible to use ricin in a chemical attack in a form absorbed through the respiratory tract. However, in the everyday life of a doctor, the most common way of poisoning with this substance is the gastrointestinal tract and accidental ingestion of castor bean seeds, especially by children. Rapid diagnosis of ricin poisoning and effective treatments are not available.

### 4.2. Fentanyl

Opioids have a wide medical application, characteristic symptoms of poisoning and quite effective methods of treatment. Due to their common use and relatively easy availability, poisoning with them, mainly due to an overdose of opioid used for therapeutic purposes, is quite common in practice, and if not recognized quickly enough, they lead to severe hypoxia, multiorgan failure and death of the patient.

### 4.3. TCDD

The use of TCDD as a poison, despite its high potency, did not directly lead to the death of any of the poisoned persons whose cases are described in the literature. The relatively long (several weeks) period of acute nonspecific symptoms may, however, prevent the poisoned person from functioning normally—in the case of Viktor Yushchenko it was an election campaign. In population, the probability of isolated poisoning with dioxin-like compounds is very low. Most often, in acute poisoning, other xenobiotics are responsible for clinical manifestation and mortality.

### 4.4. Polonium Isotope ^210^Po

Poisoning with this element is difficult to diagnose due to nonspecific symptoms. Initially, it manifests itself with problems with the digestive system, such as nausea, vomiting, diarrhea and abdominal pain. These symptoms are usually associated with food poisoning. Other symptoms such as hair loss and bone marrow depression have been associated with cytotoxic treatment, which also makes differentiation difficult. Polonium-210 poisoning can be similar to thallium poisoning, which also causes hair loss, anemia and leukocytosis. However, thallium causes painful peripheral sensory neuropathy, which helps rule out its possible use [51]. There are several treatment regimens for polonium-210 poisoning, but the effectiveness of treatment depends on the degree of irradiation and the time elapsed after exposure.

### 4.5. VX

Kim’s case identifies the VX as one of the most effective chemical weapons. Knowledge about its synthesis is widely available, and the necessary laboratory facilities can be organized privately. This is evidenced by the activity of the Aum-Shinrikyo sect, which was the first to use VX for criminal poisoning in the 1990s [15,82]. A patient’s postexposure condition can worsen within minutes, so an appropriate response is essential for survival. In the case of laboratory tests and the detection of toxic agents, it is worth using mobile sets for the rapid determination of cholinesterases and detectors of pesticides and combat gases [73,83]. They are commercially available, and their presence in critical infrastructure such as government buildings would speed up the time it takes to administer the antidote. Regular education of medical personnel is also essential. Criminal poisoning is extremely rare, and the risk of making a mistake—as in the case of too low a dose of atropine given to Kim—is high [14].

### 4.6. Novichok

There is still little reliable and publicly available information about compounds from the Novichok group. Most of the reports about them come from a single source, which does not provide adequate reliability (and sometimes even contradicts the studies performed), and the fact that all data about these substances are diligently guarded makes it still a very difficult challenge to determine their properties, toxicity and metabolism. Consequently, the diagnostic and therapeutic algorithms also cannot be precisely adjusted, resulting in a high risk of death for the exposed patient. The issue of poisoning prevention also remains unsolved. To date, a way to prevent Novichok poisoning has not been developed. The only options today are not to leave one’s belongings unattended and not to take liquids and food from an unknown source, although, as the cases of known poisonings described in this publication show, this, too, may not be sufficient.

## 5. Conclusions

Contemporary poisons used in political crime poisonings are based, to a greater extent than in the past, on the use of synthetic substances from the group of organophosphorus compounds and radioactive substances. The possibility of proper and effective treatment is the result of many factors, including the possibility of quick and competent rescue intervention, reliable detection of the substance and its metabolites in evidence and biological material (usually using tandem mass spectrometry) and the possibility of using an antidote. Contrary to other criminal poisonings, potential victims-politicians and their environment most often are fully aware of the threat to life and health, which may affect quick and targeted diagnostics and effective treatment. Prevention should include three elements: (1) the availability of simple, quick tests in critical infrastructure, (2) regular education of medical personnel, simplified to the patterns of conduct within the first hours after the poisoning (this way crucial time will be gained to acquire and use specialized knowledge) and (3) securing drug reserves in forms with a long due-date, together with kits necessary for quick decontamination in the event of a mass incident.

## Figures and Tables

**Table 1:** Properties of pharmacologically active substances facilitated criminal poisoning [1,2].

Substance Properties	Estimated Likelihood of Use in		Effect
	Acute Poisonings	Chronic Poisonings	
Tasteless and colorless	high	high	Possibility of an unnoticed administration.
Well soluble in water	high	high	Easy administration, quick absorption and distribution.
Well soluble in fats	high	very high	Possible accumulation in the fatty tissue, as well as slow release from it.
Delayed effect onset	high	very high	Impedes detection of the perpetrator and assessment of true time and place of intoxication.
Unusual and difficult to detect	very high	very high	Impedes crime detection.
Low lethal dose	very high	very high	Facilitates poisoning by making a low dose necessary to cause death.
Easy access to the substance	low	high	Facilitates crime.
Chemically stable	high	very high	Facilitates storage and transport of the poison.
Quickly degradable after death	high	high	Impedes identification of the poison and the cause of death.
Occurring naturally within the body	high	high	Does not arouse suspicion in case of detection.
Occurring naturally in the burial place	low	low	Presence within the corpse does not arouse suspicion of poisoning.

The aim of the study was to review selected cases of political criminal poisoning in the years 1978–2020. It also resulted in an attempt to answer the question of whether it was possible to quickly detect a toxic substance and undertake an effective treatment.

**Table 2:** Description of political criminal poisonings in the years 1978–2020.

Poison, Victim of Poisoning (Time, Place and Died/Survived after Poisoning)	Case Description	Symptoms of Poisoning, Treatment Undertaken, Results of Autopsy
**Ricin,** **Georgi Ivanov Markov** (11 September 1978, London, UK, died) [2,6,7]	A month before his death, while visiting a friend in Germany, Markov remembered an anonymous phone call (three months ago) that had threatened him with death if he continued to write broadcasts for Radio Free Europe. The last script Markov prepared for this radio was a text from July 1978—“The Mind Under House Arrest”, in which he accused Bulgarian radio commentators of cowardice and inability to express their own opinions. On 7 September 1978 (the 67th birthday of Bulgarian leader Todor Zhivkov), at circa 1.30 PM, Markov was waiting at the bus stop at the southern end of the Waterloo Bridge. Suddenly he felt pain in the back of his right thigh. As he turned, he saw a man bending down to get a dropped umbrella. The stranger, with a foreign accent, apologized to him for his clumsiness, then took a cab away. At first, Markov didn’t notice the whole thing until someone in the office told him about the red stain on the back of his pants. When Markov looked at the leg, he saw a red dot on his thigh. He assumed that the change would soon disappear and paid no attention to it.	Markov felt weakness 5 h after the incident. Then there was: fever and vomiting. The next day, the patient was admitted to St. James in Balhama. He had a high fever at the time and complained of abdominal pain, vomiting and diarrhea. After the examination, he was found to have enlarged, painful lymph nodes in his right groin, and a swelling of about 6 cm in diameter was observed on the posterior surface of the right thigh. The next day, the patient’s condition deteriorated—heart rate increased to 160 bpm, blood pressure dropped and increased sweating appeared. Symptoms indicated the development of septic shock. In morphology, an increase in leukocytosis was observed. Further symptoms were diuresis stopped and blood appeared in the vomit. The ECG (performed on 11 September 1978) revealed a complete atrioventricular block with numerous ventricular beats. On the same day, the patient had a cardiac arrest. During the autopsy performed on 12 September 1978, only a small mark on the back of the thigh was found. During internal examination, pulmonary edema resulting from heart failure, mild fatty liver, hemorrhagic necrosis of the small intestine and hemorrhagic lymph nodes in the right groin were found. Necrosis has also been observed in the testes, pancreas, and inguinal lymph nodes. Microscopic examination revealed small hemorrhagic foci in the myocardium. During the autopsy, a small metal ball with a diameter of about 1.5 mm was found, which fell out of the fragment of the thigh collected for examination. Two holes were drilled in the ball—one was open and the other was blind. It was found that the holes would be able to hold about 500 mg of a substance that could have contributed to Markov’s death. Already during the autopsy, it was suspected that the cause of death was not septic shock but poisoning with the toxin placed in the ball.
**Fentanyl,** **Khaled Meshal** (25 September 1997, Amman, Jordan, survived)	Khaled Mashal was a leader of the Palestinian political-military organization Hamas, which had been battling Israel for many years (especially in the area of Gaza Strip). Numerous suicide bombings conducted by the Hamas fundamentalists had led to the order of killing the Palestinian politician given by the authorities of Israel. On 25 September 1997 Meshal was attacked by two men from an Israeli intelligence—Mossad. The attack was carried out by spraying a toxic substance into the victims’ ear.	One of the first symptoms experienced by the victim was tinnitus and a sensation of an electrical current going through his body. After approximately 2 h he started feeling nauseous, short of breath and started vomiting soon after. He was admitted to a hospital where, due to acute respiratory failure, he needed a mechanical ventilation. Meshal’s condition improved only after administering the antidote (naloxone) provided by the Israeli authorities.
**TCDD** **(2,3,7,8-tetrachlorodibenzo-*p*-dioxin),** **Viktor Andriyovych Yushchenko** (5 August 2004, Kyiv, Ukraine, survived) [2]	Viktor Yushchenko, a political activist in opposition to pro-Russian political groups, and a candidate for president of Ukraine in the presidential election. On the day of the incident after dinner with Igor Smeshko (head of the Security Service), Yushchenko felt bad. Viktor Yushchenko’s wife said that on the evening of poisoning she felt a strange taste of “medicine” when kissing her husband. Viktor Yushchenko survived. Dose of absorbed poison estimated at approx. 1.5–2.5 mg TCDD.	Within hours of the exposure, Yushchenko developed nausea, vomiting and abdominal pain. On 6 August 2004, the patient was diagnosed with food poisoning. Due to the lack of improvement after treatment, the patient was transferred to the Rudolfinerhaus clinic in Vienna. In the clinic, Yushchenko was diagnosed with acute edema pancreatitis, toxic liver damage and gastrointestinal ulceration. After a week of treatment, the patient returned to Ukraine. Unfortunately, after two weeks, due to back pain and “half palsy of the face”, the patient had to be returned to the Viennese clinic. Approximately 3 weeks after the poisoning, the patient developed a characteristic chloracne on his face. Only this symptom led doctors to suspect dioxin poisoning. This suspicion was finally confirmed on 12 December 2004. The concentration of TCDD in the blood of Viktor Yushchenko was 50,000 times higher than the general population’s acceptable level of the above-mentioned substance in blood. During the treatment, attempts were made to accelerate the elimination of the xenobiotic by using orally olestra—an unabsorbed fat substitute. As a result of the measures taken, the half-life of TCDD in the patient’s body was reduced from approx. 7–8 years to approx. 15.4 months. The skin changes resolved within 3–5 years.
**Isotope of polonium** **^210^Po,** **Jasir Arafat** (11 November 2004, Percy, France, died) [2]	Yassir Arafat was the president of the Palestinian Authority and leader of the liberation movement. Before his death, he was in solitary confinement at the Palestinian Authority headquarters in Ramallah for about 3 years. On 12 October 2004, about 4 h after a meal, he developed severe nausea, vomiting, abdominal pain and then watery diarrhea. On 29 October 2004, the patient was transferred to the French Percy Military Hospital in Clamart, where he was diagnosed with enteritis. Urine tests were negative for gamma-emitting radionuclides. On 11 November 2004, Arafat died of a cerebral hemorrhage. An autopsy was not performed. It was only after the murder of Litvinenko (2006) that it was suggested that the cause of Yassir Arafat’s death could have been polonium-210. As the biological samples collected during the patient’s hospitalization were destroyed in 2008, an exhumation was performed on 27 November 2012, on the basis of which it was found that the hypothesis of Arafat polonium-210 poisoning cannot be fully rejected.	About 4 h after the meal, the patient complained of nausea, vomiting, abdominal pain and watery diarrhea. The studies revealed: presence of megakaryocytes and an increased number of macrophages, enteritis, severe disseminated intravascular coagulation (DIC) with marked thrombocytopenia, cholestatic jaundice and renal failure. Arafat’s death was due to intussusception following a cerebral hemorrhage.
**Isotope of polonium** **^210^Po,** **Aleksander Litwinienko** (1 November 2006, London, UK, died) [2]	Alexander Litvinenko (pseudonym Edwin Carter) was a former KGB lieutenant colonel. In 2000, he obtained asylum in Great Britain and began working as a consultant in the British intelligence services. On 1 November 2006, Litvinenko met with a former KGB agent. He felt bad after eating sushi. He developed stomachache, vomiting and diarrhea. On 3 November 2006, he presented himself to the Emergency Department of Barnet General Hospital under the pseudonym Edwin Carter. On 10 November 2006, the presence of the toxin *Clostridium difficile* was identified. In the course of presenting the diagnosis, the patient revealed his true identity and suggested that he may have been poisoned. On 17 November 2006, the results of the screening showed a slightly elevated thallium concentration, but below the toxic dose. Despite this, the patient was treated. Alexander Litvinenko died on 23 November 2006 as a result of cardiac arrest. Shortly after his death, urine samples collected from him on 22 November 2006 were analyzed, and poisoning with polonium-210 was found.	Day: 1–3—stomachache, vomiting, diarrhea, upper abdominal tenderness, slightly elevated urea levels. Day 11—the appearance of a fever. Day 13—alopecia, mucositis, progressive cytopenia. Day 18—jaundice with normal levels of alanine transaminase, bloody vomiting. Day 19—arrhythmias, fever, elevated markers of inflammation. Day 20–22—rapid deterioration in kidney function. Day 22—rash, progressive metabolic acidosis, oliguria, hypothermia, cardiogenic shock, impaired consciousness, cardiac arrest twice. Day 23—cardiac arrest, death. Autopsy results: blood-stained fibrous pericarditis, pleural effusion associated with bilateral pneumonia, ascites, generalized tissue autolysis in most organs, brain unchanged. Estimation of the concentration of polonium-210—4400 MBq.
**organophosphorus compound VX,** **Kim Jong-nam** (13 February 2017, Kuala Lumpur, Malaysia, died) [2]	Kim Jong-nam, as the eldest son of Kim Jong-Il, was originally being prepared for his successor [8]. As a result of a diplomatic scandal, his half-brother Kim Jong-Un seized power in North Korea and ordered his relative killed [9,10]. Jong-nam was poisoned at Kuala Lumpur Airport. The killing was carried out by two women. Within 7 s, each applied a poisoned handkerchief to his face [11,12,13].	After the attack, Kim reported to an airport medical facility. There he observed: trembling hands, hyperhidrosis and weakness [11]. During a physical exam, Jong-nam showed symptoms of cholinergic syndrome [14]. He then received 1 mg of atropine and adrenaline. Due to respiratory failure, he was intubated and connected to a ventilator. The death occurred 20 min after the attack. The autopsy report is probably not available to the public [15].
**Novichok-type organophosphorus compound,** **Sergei Skripal** (4 March 2018, London, UK, survived) [2]	Sergei Skripal became famous as a Russian military intelligence officer who in the 1990s decided to become a double agent for the British intelligence services. He obtained secret information while working at increasingly senior levels of the GRU and later also in government institutions. In December 2004, he was arrested and convicted of treason by a Moscow military court. In 2010, as part of a spy exchange between the Russian Federation and the United Kingdom, Skripal was transported to England, and he settled in Salisbury. Despite being exposed, he continued to cooperate with Western intelligence agencies. On 4 March 2018 Sergei Skripal and his daughter Yulia were found unconscious on a park bench in Salisbury. An investigation by British services found that they were poisoned by two GRU officers, Anatoly Chepiga and Alexander Mishkin, who sprayed the Novichok agent on the doorknob and front door of Skripal’s home. Traces of Novichok were also found in the pub where the Skripals spent the afternoon. The perfume bottle used by the agents as a container for the poison was then dumped in a container for donations to the needy. It was found by a random British man, Charlie Rowley, and given to his partner Dawn Sturgess. The woman died shortly after spraying her wrists with the substance contained therein. Despite wearing a full protective suit, a police officer searching Skripal’s home was admitted to hospital in serious condition. Investigators later determined that the bottle used in the incident contained enough Novichok to kill thousands of people. Specialists identified the agent used as most likely A-234.	According to the testimony of witnesses present at the scene, Sergei and Yulia Skripal were unconscious. Foam was coming out of Yulia’s mouth. On admission to hospital, the condition of both was described as critical. Due to the characteristic symptoms, treatment with atropine was immediately started, and anticonvulsants were included. The patients’ condition improved gradually. Yulia Skripal left the hospital on 9 April 2018, and her father on 18 May 2018. After the inspection, access to the interior of Skripal’s house was secured. Scaffolding was erected around the building, on which special protections were placed. It took about 4 months to strip the roof, clean the house and rebuild it. All vehicles involved in the incident, including ambulances and police cars, were disposed of and buried in the Cheltenham landfill. It has long been questioned how Skripals managed to survive the attack despite being exposed to such a high dose of A-234. Both one of the Novichok developers and other scientists agreed that weather played a significant role in the case. On the day of the Skripals’ poisoning in Salisbury, it was humid and foggy, and humidity reduces the harmfulness of this type of poison. It is worth noting that the weather conditions only hindered the poison’s absorption, not its toxicity per se. Skripals were also inadvertently helped by the agents who tried to liquidate them. To help Novichok stick to various surfaces, including skin, it was mixed with a gel-like substance. This agent slowed down the absorption of the poison into the body which made it possible to apply effective treatment.
**Novichok-type organophosphorus compound,** **Alexei Navalny** (20 August 2020, Tomsk, Russia, survived)	Alexei Navalny, leader of the Russian opposition, chairman of the Russia of the Future party and founder of the Foundation for the Fight against Corruption, has for many years strongly criticized the policies pursued by Vladimir Putin and his United Russia party. On 20 August 2020, Navalny boarded a flight from Tomsk to Moscow. During the flight his well-being suddenly deteriorated. Therefore, the pilots decided to make an emergency landing in Omsk and transport the patient to a local hospital. Navalny was successfully transported to the Charité hospital in Berlin on 22 August 2020. Navalny’s subsequent investigation revealed that Novichok had been sewn by FSB agents into the seams in his underwear left in his hotel room.	The first symptoms noticed in Navalny were pallor, intense sweating, drooling, vomiting and loss of consciousness. Doctors at the Omsk hospital quickly suspected poisoning with an agent from the acetylcholinesterase inhibitor group. Navalny was put into a coma, intubated, mechanically ventilated. The patient was also administered atropine. On admission to Charite Hospital, the patient was additionally observed to have hypothermia, bradycardia, impaired trunk reflexes, exaggerated tendon reflexes and pyramidal symptoms. Initial investigations confirmed that the patient had been poisoned with a paralytic agent from the cholinesterase inhibitor group. Laboratory evidence confirmed reduced butyrylcholinesterase levels in the patient’s blood. Based on samples sent to a specialized unit, Novichok was confirmed in blood, urine and on Navalny’s skin. The treatment included atropine and obidoxime. The patient left the hospital on his own after 33 days, including 24 days of respiratory therapy.

**Table 3:** Selected poisonous substances used for criminal purposes in the years 1978–2020 on a political background, their physicochemical and toxicological properties.

Toxic Substance (CAS)	Physicochemical Properties	Toxicological Properties		Refs.
		Lethal/Incapacitating Dose [mg⋅min^−1^⋅m^−3^]	Time of Death [h]	
**Ricin** (9009-86-3)	White powder. It can be prepared in liquid/crystalline form.	Lethal dose: p.o. 20–30 mg/kg (rats) p.o. 15–35 mg/kg (mice) p.o. 1–20 mg/kg (human), about 5–10 castor bean seeds i.v. 3 to 5 µg/kg (mice) s.c. 22 µg/kg (mice) aero. 5–15 μg/kg (human) i.m. 0.8 μg/kg (guinea pig) no data on lethal doses in humans	Several dozen hours (with p.o. possible delay in absorption up to 5 days)	[16,18,19,20]
**Fentanyl** (437-38-7)	Crystal-like solid, moderately water-soluble	Lethal dose: i.v. 2.91 mg/kg (mice) p.o. 368 mg/kg (mice) p.o. 18 mg/kg (rats) s.c. 62 mg/kg (mice) s.c. 1.5 mg/kg (rats) no data on lethal doses in humans		[16,21]
**TCDD** (108-88-3)	Crystalline, colorless solid, soluble in organic solvents, hydrophobic	Lethal dose LD_50_: p.o. 2 μg/kg (guinea pig) p.o. 70 μg/kg (king macaque) p.o. 22–45 μg/kg (rats) p.o. 5051 μg/kg (hamster) no data on lethal doses in humans	From several days to several weeks	[16,22,23]
**Isotope of polonium ^210^Po**	Radioactive metal, soluble in water, forming simple salts in dilute acids	Lethal dose LD_50_: 50 ng (oral suspension) 10 ng (inhalation)	From 2–3 weeks after the onset of symptoms	[16,24]
**organophosphorus compound VX** (50782-69-9)	Amber to transparent oily liquid, slightly soluble in water	Lethal dose (predicted): LCt50 30 (air-cutaneous) LCt50 7 (aerosol) Incapacitating dose (predicted): ECt50 25 (air-cutaneous) ECt50 10 (aerosol)	A few to several minutes—bronchospasm	[16,25,26,27]
**organophosphorus compound Novichok** **(no clear identification of the compound)**	Liquid, fine powder, no details available	For A-230 (estimated for human): LC_t50_—1.9–3 mg-min/m^3^ LD_50_—7.5 × 10^−4^ − 0.002 g/70 kg body weight Dla A-232 (estimated for human): LC_t50_—7 mg-min/m^3^ LD_50_—0.035 g/70 kg body weight Dla A-234 (estimated for human): LC_t50_—7 mg-min/m^3^ LD_50_—0.035 g/70 kg body weight		[16,28]

p.o.—*per os*, i.v.—*intravenosa*, i.m.—*intramuscularis*, s.c.—*subcutanea*, LC—lethal concentration, LD—lethal dose.

**Table 4:** Scheme of aid and treatment of poisoning with polonium-210 by the oral route [51].

No.	Stages during Treatment of ^210^Po Intoxication by the Oral Route
1	Gastric lavage—effective up to an hour after ingestion; reduces the risk of absorption.
2	Antiemetics, intravenous fluids, analgesics.
3	Treatment of bone marrow failure—application of colony simulations; in severe thrombocytopenia and anemia: (a) GSF—granulocyte colony stimulating factor. (b) Pegfilgastrim—a factor that stimulates the formation of neutrophils. (c) Stem cell transfusion—not used in patients with complete bone marrow failure.
4	Chelation therapy—reduces the retention of radiation in the blood and organs but increases retention in the kidneys (sometimes also in the liver and brain). (a) according to the recommendation of United States, the National Council on Radiation Protection and Measurements, Dimercaprol (2.5 mg/kg) should be administered intramuscularly: 4 times a day for 4 days. 3 consecutive days twice a day. 10 consecutive days once a day. (b) according to the recommendation of Radiation Event Medical Management, Dimercaprol should be administered intramuscularly (2.5 mg/kg or less): 2 days every 4 h. Then a diagram as above. (c) according to the Recommendation of Defense Research and Development Canada, Dimercaprol should be administered intramuscularly: Test dose (to check the sensitivity of the body)—75 mg. 300 mg every 4 h for 3 days.
5	Palliative care—In case of high irradiation—relieving symptoms and stress

**Table 5:** Comparison of Polish and American standards in the treatment of poisoning with paralytic and convulsive factors in adults [67,68].

Hospital Treatment of Poisoning with Paralytic and Convulsive Factors in Adults			
Recommendations	POLISH—As a Procedure in the Case of Cholinergic Syndrome	AMERICAN—Specific for Poisoning with a Substance from the Paralytic and Convulsive Group	
Division into age groups	no	yes	
Initial activities and their sequence	Monitoring of heart function and breathing. Use of oxygen therapy. Pharmacotherapy.	ABCDDS: Follow the ABC rules. D—decontamination. Ds—drugs—basic pharmacotherapy.	
Degree of poisoning	No separation	Mild/medium	Heavy
Basic treatment	Atropine 1–5 mg i.v. D—decontamination, repeat the dose every few minutes so that suctioning of the bronchial contents is not needed more than once an hour. Oximes: 250 mg every 4–6 h or Pralidoxime 30 mg/kg every 4–6 h.	Atropine 2–4 mg i.v./i.o./i.mD—decontamination then administer a dose of 2 mg at intervals of 5–10 min until exudates are gone and breathing is comfortable or airway resistance returns to normal. Pralidoxime 600 mg i.v./i.o./ i.m.	Atropine 6mg i.v./i.o./i.m.—then administer a dose of 2 mg at intervals of 2–5 min until the exudation is gone and breathing is comfortable or the airway resistance returns to normal. Pralidoxime 1800 mg i.v./i.o./i.m. Midazolam 10 mg i.v./i.o./i.m. OR Diazepam 10 mg i.v./i.o./i.m. OR Lorazepam 6 mg i.v./i.o./i.m.
Symptomatic treatment	In convulsions or overstimulation: diazepam 10 mg i.v., repeat as needed	In convulsions, additional doses of benzodiazepines or barbiturates may be used. In bronchospasm, if the desired effect has not been obtained with atropine, inhalation/nebulization with ipratropium and one of the following beta-agonists can be used: SABA—albuterol 2.5 mg/3 mL; terbutaline 1 mg/mL LABA—formoterol 0.02 mg/2 mL, salmeterol (only inhaled) 0.05 mg. If the above treatments are unsuccessful, you can give systemically 1–2 mg/kg methylprednisolone Siarczan magnezu 2 g i.v.	

i.o.—*intraossea*, i.v.—*intravenosus*, i.m.—*intramusculare*.

## Data Availability

Not applicable.
